# Primary Technology Enhanced Care Home HbA1c Testing (PTEC HAT) programme: a feasibility pilot study in Singapore

**DOI:** 10.1186/s12875-024-02373-w

**Published:** 2024-04-23

**Authors:** Shilpa Tyagi, Gerald Choon-Huat Koh, Eng Sing Lee, Kah Pieng Ong, Roy Heng, Lian Hwa Er, Evonne Oh, Valerie Teo, David Wei Liang Ng

**Affiliations:** 1 MOH Office for Healthcare Transformation (MOHT), Singapore, Singapore; 2 Saw Swee Hock School of Public Health, National University of Singapore, Singapore, Singapore; 3 National Healthcare Group Polyclinics, Singapore, Singapore

**Keywords:** Diabetes, Tele-monitoring, HbA1c testing, Point-Of-Care testing

## Abstract

**Background:**

Considering time-consuming, cost-related limitations of laboratory-based HbA1c testing and follow-up clinic visits for diabetes management, it is important to explore alternative care models which incorporate point-of-care testing for HbA1c to monitor glycaemic control and related management.

**Methods:**

Therefore, we adopted an implementation perspective to conduct one group pre- and post-intervention feasibility pilot assessing feasibility, acceptability and satisfaction with conducting home HbA1c test by patients with type 2 diabetes coupled with telemonitoring and teleconsultations (i.e., the Primary Technology Enhanced Care (PTEC) Home HbA1c Testing (HAT) Programme) in Singaporean primary care setting. The secondary objective was to compare the HbA1c, blood pressure and primary care visits at the end or during intervention, vs. 6 months before. Adult patients with type 2 diabetes with HbA1c ≤ 8% without any diabetes complications and having phone compatibility were recruited. Data was collected via patient self-reports and electronic medical records extraction. While summary statistics and paired t-test were computed for quantitative data, open-ended feedback was analysed using content analysis.

**Results:**

A total of 33 participants completed the intervention out of 37 (33/37 = 89%) recruited from 73 eligible (37/73 = 51%). Most were either 51 to 60 years old (46.9%) or more than 60 years (37.5%), with more males (53.1%) and majority Chinese (93.8%). Majority (81.3%) felt that home HbA1c testing was beneficial with most commonly reported benefit of not having a clinic visit. A key finding was the average of diabetes-related visits being significantly lower post-intervention with comparable HbA1c values pre- and post-intervention. The most commonly reported challenge was using Bluetooth to transmit the reading (43.7%), followed by having too many steps to remember (28.1%). While participants reported being overall satisfied with the intervention, only 22% were willing to pay for it.

**Conclusion:**

Our findings support home HbA1c testing by patients coupled with telemonitoring and teleconsultations. Following are practical recommendations for the implementation scaling phase: offering PTEC HAT Programme to suitable patients who are self-motivated and have adequate digital literacy, provision of adequate educational and training support, sending reminders and exploring enabling manual submission of HbA1c readings considering Bluetooth-related challenges.

**Supplementary Information:**

The online version contains supplementary material available at 10.1186/s12875-024-02373-w.

## Background

Diabetes is a chronic disease afflicting many countries, Singapore notwithstanding, and its prevalence is projected to increase, associated with increased healthcare costs [1, 2]. Glycated haemoglobin (HbA1c), a well-established surrogate of glycaemic control and a strong marker of diabetes complications [3], is currently a laboratory-based test and only available in healthcare setting in Singapore requiring patients with sub-optimal control to visit primary care clinics multiple times a year for monitoring and clinical management of their diabetes [4]. Not only is laboratory-based HbA1c test time-consuming and costly, it may be inconvenient for the patient to visit the healthcare setting multiple times [5]. Hence, it is important to explore alternative care models which offer convenience of timely HbA1c testing and efficient follow-up.

Point-Of-Care (POC) testing, defined as the “ability to move testing closer to the patient” [6], is typically designed to be conducted in an office, bedside or treatment room with the main advantage being rapid availability of the results and subsequent immediacy of management [7, 8]. With ample existing literature reporting POC testing in different areas of clinical medicine, the main challenge identified is inadequate technology adoption to achieve the intended health outcome highlighting the need for accompanying change in care processes to facilitate POC testing integration with usual care [6]. Similar findings are reported for HbA1c POC testing in the primary care setting with the challenges highlighted related to time constraints faced by clinicians, high workload, and inertia to adopt a new intervention [7]. Thus, there is a need to explore alternative approaches to incorporate POC testing for HbA1c in the management of patients with diabetes in the primary care setting.

While the POC test for HbA1c is usually performed in a clinical setting by a trained professional, there is evidence to support the accuracy of performing the HbA1c POC test by lay users [9–11], lending support to the potential implementation of home HbA1c testing by patients along with other complementary features to introduce an alternative care model in the primary care setting. Considering there is evidence supporting incorporation of communication with healthcare team as part of a telehealth intervention that results in an improvement in HbA1c [12–14], one possible care model worth exploring is the coupling of self-test for HbA1c by patients at home with transmission of results to the care team and follow-up teleconsultations. Not only does this care model empower the patient in self-management of his/her diabetes, but it also affords the immediacy of therapeutic advice, further obviating the need to visit the healthcare setting. A randomized controlled trial involving patients with diabetes conducted in a Western setting involving mainly people of Latino origin established the efficacy in a controlled experimental setting of home HbA1c testing using a POC kit and phone consultation by the clinician [13]. Not only was there improvement in glycaemic control but there was also a significant increase in physical activity and perception of adherence to diabetes self-care practices in the intervention group as compared to the control group. With the intervention efficacy established in a controlled setting, it is important to translate this evidence from a research setting into practice change in real-world setting. To enable this knowledge transfer and bridge the knowledge-practice gap, we adopted an implementation perspective to conduct a pilot to assess the feasibility, acceptability and satisfaction with conducting home HbA1c test by patients with type 2 diabetes coupled with telemonitoring and teleconsultations in the primary care setting in Singapore. Additionally, the secondary objective was to compare the HbA1c, blood pressure and primary care visits (all-cause and diabetes-related) *at the end or during* the intervention, vs. 6 months *before* participation in the intervention.

## Methods

### Setting

**Fig. 1:**
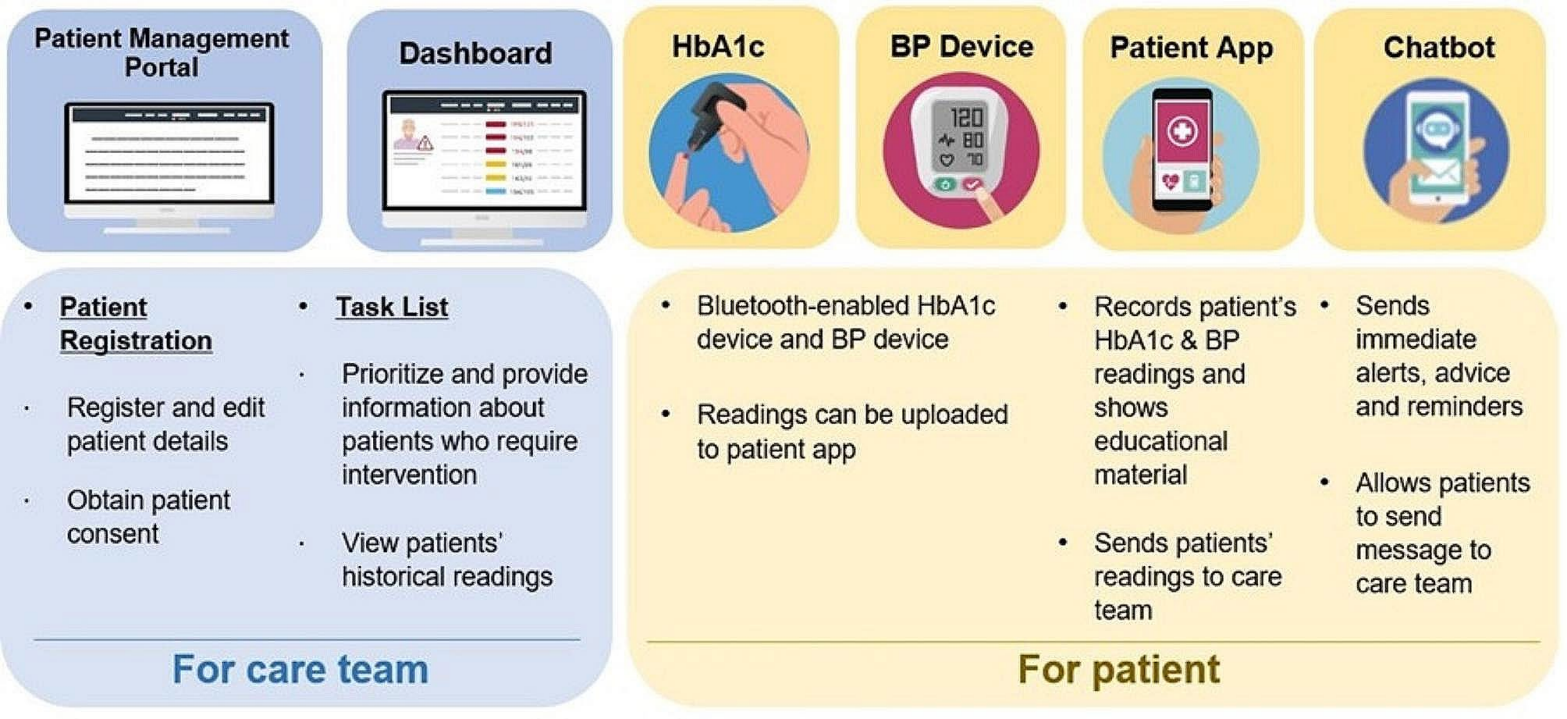
Overview of the PTEC HAT Programme

The Primary Technology Enhanced Care (PTEC) Home HbA1c Testing (HAT) Programme, a complex intervention, was conceptualized based on evidence synthesis and practice insights by adopting a co-development approach between the team at the Ministry of Health’s Office for Healthcare Transformation (MOHT), the Information Technology (IT) partners and the care team from the partnering public primary care clinic from the National Healthcare Group Polyclinics (NHGP) [15] in Singapore. The Singapore primary care system comprises private and public primary care clinics. The public primary care clinics, also known as “polyclinics”, are grouped under three nationwide clusters, with NHGP being one of them. Polyclinics are one-stop healthcare centres, which provide subsidized primary care services, including medical management, preventive care and health education [16]. Figure 1 presents an overview of the PTEC HAT Programme, which was implemented as a feasibility pilot in Singapore between July 2021 to September 2022. The care team at the participating NHGP polyclinic was provided with a patient management portal via which they could register interested patients and obtain informed consent, prioritize and provide care as needed via an interactive task list and view patients’ historical readings. For the patients, the PTEC HAT Programme provided a Bluetooth-enabled HbA1c device and Blood Pressure (BP) device (if the patient had hypertension along with diabetes). The patients were asked to record their HbA1c and BP readings as per the recommended frequency (for HbA1c once every 3 to 6 months, for BP once a week) via the patient-facing smartphone app, which also showed educational material to the patients. The readings from Bluetooth-enabled HbA1c device were directly detected by the PTEC HAT smart phone app and automatically transmitted to the care team, who could access them via the dashboard function on patient management portal. Additionally, the patients received immediate alerts, advice and reminders via an in-app chatbot which also allowed the patients to send messages to their care team.

### Participants

Longitudinal one-group pre- and post-study design was adopted as the primary objective was to ascertain the patient’s experience and ability to perform a home HbA1c test. Randomization was not practical considering the real-world setting and funding constraints. Sample size estimation was not done considering the feasibility scope of the current pilot study. The recruitment continued till March 2022 in the selected polyclinic in Singapore. Patients were recruited by the consulting physician after assessing eligibility based on the following inclusion criteria: aged 21 to 80 years, having type 2 diabetes with HbA1c ≤ 8% without any diabetes complications or other conditions which may warrant a more frequent follow-up than 3 to 4 visits to the clinic per year, and patient’s smartphone being compatible with the PTEC HAT smartphone app. Following were the exclusion criteria: patients on insulin, cognitive impairment, pregnancy, pre-existing anaemia of any cause, history of ischemic heart disease, congestive heart failure, stroke, transient ischemic attack, atrial fibrillation and renal impairment, with complications or target organ damage or complex medical conditions (e.g., Parkinson’s Disease, dementia, etc.), who were on active titration of medications such as angiotensin-converting enzyme inhibitors/angiotensin-receptor blockers, and who were concurrently participating in another clinical study or programme involving a novel therapeutic drug/ device, at any time during the study period. After being recruited, the patients were trained by the care team on how to perform the home HbA1c test along with providing them with educational resources comprising a video tutorial and a guidebook. Since the home HbA1c test was to be performed once in 3 to 6 months as per the recommendation of the treating physician, the patients received an in-app chatbot reminder notification when they were due to perform the test. After performing the home HbA1c test and uploading their readings on the smartphone app, the patients were followed up by their care teams via teleconsultations to provide timely medical interventions.

The home HbA1c test was performed using the PTS Diagnostics’ A1CNow + device [17]. It is a POC device for HbA1c testing, which uses a drop of whole blood collected via finger prick. It is a handheld device with a measuring range of 4 to ≤ 15% HbA1c and a testing time of 5 min. The A1C Now + is National Glycohemoglobin Standardization Programme (NGSP) and International Federation of Clinical Chemistry and Laboratory Medicine (IFCC) certified and Clinical Laboratory Improvement Amendments (CLIA) waived in the USA. In addition, it was Health Science Authority (HSA) approved in Singapore on 18 April 2018 as a Class B In Vitro Diagnostic (IVD) medical device (device registration number – DE0500281). The product has a shelf-life of 15 months and is stored at 4–8 °C. It must be thawed for at least 30–45 min before use. Even when it is exposed to room temperature for 10 days; the shelf life of the item is 120 days minus 10 days, hence it will have 110 days good for use [17]. The accuracy of A1CNow + system has been reported in the literature [18]. Henceforward, A1CNow + kit or test would be referred as the HAT kit or HAT test.

### Data collection

#### Variables

Data collected from the following modes was used for the current evaluation: (i) patient self-reported survey conducted at baseline and after completion of at least one home HbA1c test, and (ii) vital parameters captured during clinic visit (HbA1c and BP readings), utilisation and comorbidity data extracted from the electronic medical records.

##### Questionnaires

There were two surveys conducted online using FormSG [19], a secure national portal for creation and completion of surveys, one at baseline and another after completion of at least one home HbA1c test. For the baseline survey, socio-demographic information and answers to the following diabetes instruments were collected: The revised Michigan Diabetes Knowledge Test (DKT) [20], Diabetes Empowerment Scale (DES-SF) [21] and the Understanding Component of Diabetes Care Profile (DCP) [22]. For the user experience survey, since there was no pre-existing instrument that could be adapted for use, information was captured using questions developed by the project team based on preliminary feedback from a user experience workshop conducted by the project team to prepare for this feasibility pilot. The questions included feedback on the experience of doing the HbA1c test at home, perceived barriers, difficulties, benefits of doing the home HbA1c test and satisfaction with the PTEC HAT Programme. Additionally, open-ended questions were included to get feedback on which aspects were liked or disliked by the patients along with feedback on potential improvements. Please refer to Additional File 1 for the user experience survey questions.

##### Utilisation and Comorbidity Data from Electronic Medical records

For utilisation, polyclinic visits (both all-cause and diabetes-related) were extracted for six months pre-enrolment (excluding the visit of enrolment) and six months after the enrolment date in the HAT feasibility pilot. Additionally, clinic-based HbA1c and BP values six months pre- and post-enrolment date were extracted. The duration of six months was extended by another three months if no values were available over six months. Additionally, concurrent chronic conditions were extracted from coded diagnoses in the patients’ electronic medical records at the point of enrolment based on data availability.

### Data analysis

Summary statistics (for example, proportions for categorical variables, mean and standard deviation (SD) for continuous variables) were computed to describe the baseline profile of enrolled patients based on demographic characteristics, DKT, DES-SF and the understanding component of DCP scores. DKT questions were given a score of 1 for a correct answer and 0 for a wrong one. Subsequently, the total score was calculated by adding scores of individual questions and dividing by the number of questions answered (there were 17 questions but 2 may not be answered if respondent was not on insulin). The final score was converted to a percent score, with those who scored 65% and more being categorised as having good diabetes knowledge [23]. For the DES-SF instrument whose questions were all phrased positively, an item checked as “strongly agree” received 5 points, “agree” received 4 points, “neutral” received 3 points, “disagree” received 2 points and “strongly disagree” received 1 point. The numerical values for all the questions were added and the sum was divided by the total number of questions answered (*n* = 8). For the Understanding Component of the DCP scale, each question received a rating from the patient ranging from 1 (i.e., poor) to 5 (i.e., excellent). The numerical values for all the questions were added and the sum was divided by the total number of questions answered (*n* = 13). Additionally, summary statistics were computed to describe the benefits, barriers and challenges or difficulties encountered by patients while doing home HbA1c testing along with satisfaction with the PTEC HAT Programme. The paired t-test was performed to assess whether the difference between pre-intervention and post-intervention values of all-cause visits, diabetes-related visits, HbA1c (%), systolic and diastolic BP was statistically significant. The open-ended questions were analysed using content analysis [24] with summary findings presented in the form of frequency of occurrence along with several representative quotes. All statistical analysis was performed in Stata 14 [25]. 

This feasibility pilot was approved by the Institutional Ethics Board (Ref No. 2023/00230). All methods within the current study were carried out in accordance with the guidance provided by the Declaration of Helsinki and approved by the Institutional Ethics Board.

## Results

**Fig. 2:**
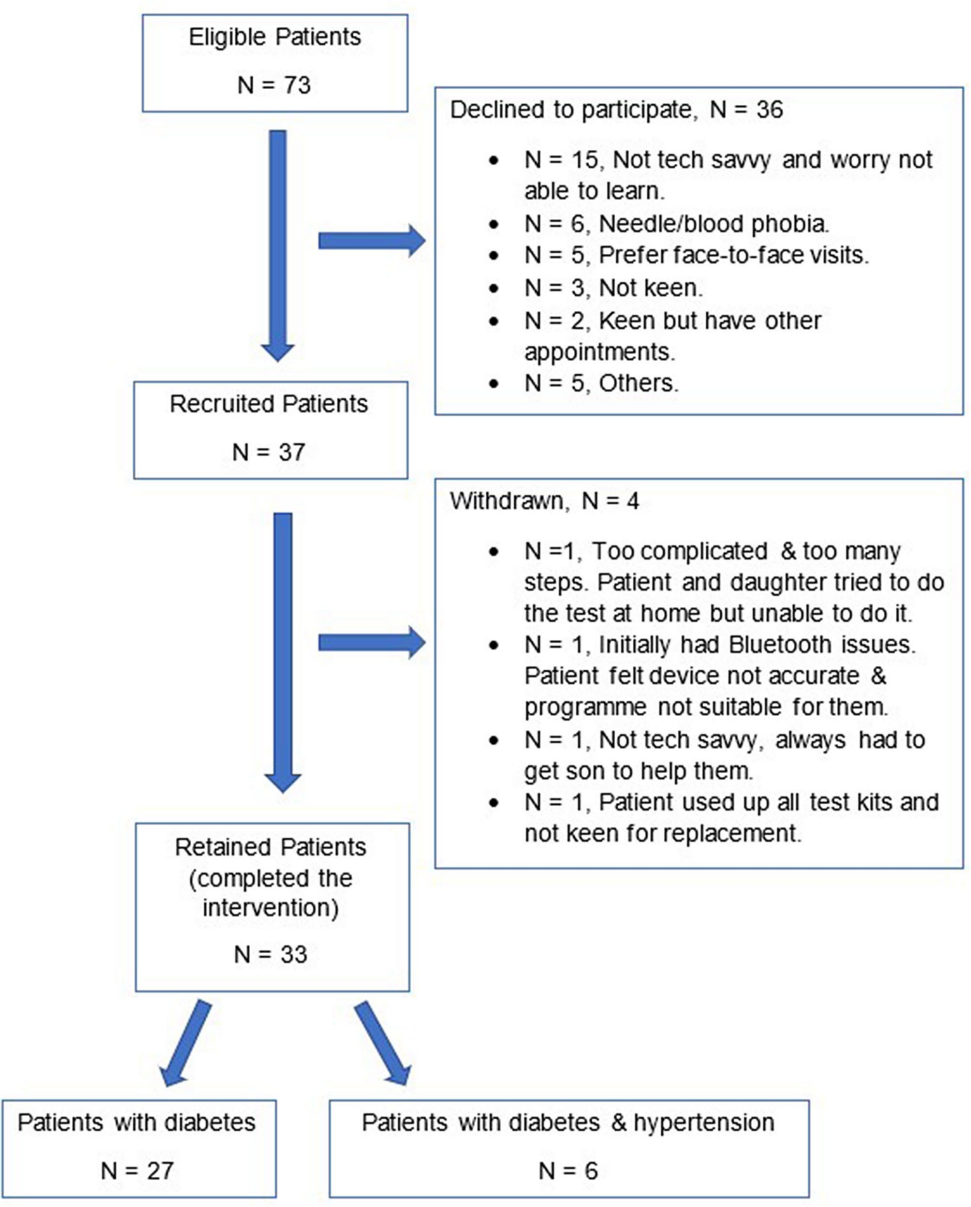
PTEC HAT Programme Feasibility Pilot Flowchart

A total of 33 patients with diabetes completed the intervention out of 37 (33/37 = 89%) recruited from 73 eligible participants (37/73 = 51%). The most common reason for declining to participate in the study was not being technologically savvy and being worried about the steep learning curve. Other reasons given were needle or blood phobia, preference for face-to-face visits (as opposed to tele-consults), having other appointments and not being keen. Please refer to Fig. 2 for further details.

### Quantitative findings

Most of the participants were either 51 to 60 years old (46.9%) or more than 60 years (37.5%), with more males (53.1%) than females (46.9%) and majority were of Chinese ethnicity (93.8%). The most common comorbid conditions with diabetes were hyperlipidaemia (81.5%) and hypertension (63.0%). The mean DKT score was 53.0, with about 15.6% (*N* = 5) having good knowledge of diabetes. (Table 1)

**Table 1:** Baseline characteristics of participants in PTEC HAT Programme Feasibility Pilot

Demographic Variable		Number (%)*
Age	21–30 years	0 (0.0)
	31–40 years	1 (3.1)
	41–50 years	4 (12.5)
	51–60 years	15 (46.9)
	More than 60 years	12 (37.5)
Gender	Male	17 (53.1)
	Female	15 (46.9)
Ethnicity	Chinese	30 (93.8)
	Malay	0 (0.0)
	Indian	1 (3.1)
	Others	1 (3.1)
Monthly household income	Less than $500	
	$500 - $999	0 (0.0)
	$1000 - $1999	0 (0.0)
	$2000 - $2999	1 (4.6)
	$3000 - $3999	3 (13.6)
	$4000 - $4999	2 (9.1)
	$5000 and above	7 (31.8)
	Don’t know	4 (18.2)
	Refused to answer	5 (22.7)
Concurrent chronic conditions	Type 2 Diabetes	27 (100.0)
	Hypertension	17 (63.0)
	Hyperlipidaemia	22 (81.5)
	Asthma	0 (0.0)
	Arthritis	1 (3.7)
	Anxiety disorder	1 (3.7)
	Allergic Rhinitis	2 (7.4)
Revised Michigan Diabetes Knowledge Test (DKT), score in %	Mean (SD)	53.1 (11.4)
	Median (IQR)	52.5 (44.7, 61.1)
	Range	33.3, 83.3
Categorical DKT^1^	Good Knowledge	5 (15.6)
	Poor Knowledge	27 (84.4)
Diabetes Empowerment Scale (DES-SF)	Mean (SD)	3.6 (0.8)
	Median (IQR)	3.3 (3.1, 4.0)
	Range	1.5, 5.0
Understanding Component of Diabetes Care Profile (DCP)	Mean (SD)	3.3 (0.8)
	Median (IQR)	3.3. (3.0, 3.8)
	Range	1.6, 5.0

*Unless otherwise stated.

^1^:Good knowledge is defined as 65% or more score on DKT.

The majority (81.3%) of the participants felt that using a HAT kit to do home HbA1c testing was beneficial with obviating the need to visit the clinic being reported as the most commonly reported benefit (68.7%). About 37.5% of participants reported barriers to using HAT kit at home. Based on their experience of home HbA1c testing using the HAT kit, about 22% of the patients were willing to pay for this service combined with teleconsultation in the future, while about 34% reported being unsure. (Table 2) More than half (59.4%) felt it was challenging to do the HAT test at home. The most commonly reported challenge was using Bluetooth to transmit the reading (43.7%). The second most frequently reported challenge was having too many steps to remember to complete the test (28.1%). (Table 3)

**Table 2:** User experience, perceived barriers and perceived benefits of Home HbA1c Testing

User Experience		Number (%)
Do you feel there are any benefits of using HAT kit at home?	Yes	26 (81.3)
Do you feel there are any barriers to using HAT kit at home?	Yes	12 (37.5)
Do you feel it is difficult/challenging to do HAT test at home?	Yes	19 (59.4)
Based on your experience of using HAT kit at home, will you be willing to pay for this service with tele-consult in future?	Yes	7 (21.9)
	No	14 (43.7)
	Unsure	11 (34.4)
**Perceived Benefits**		**Number (%)**
No need to visit the clinic	Yes	22 (68.7)
Save transport related cost and time	Yes	17 (53.1)
It is more convenient	Yes	13 (40.6)
Do not have to trouble my caregiver to assist me with clinic visit	Yes	0 (0.0)
I am more aware of my readings	Yes	8 (25.0)
I am more motivated to manage my diabetes	Yes	3 (9.4)
Tele-consults with care team to get feedback on my HAT test readings	Yes	1 (3.1)
Not applicable	Yes	6 (18.7)
**Perceived Barriers**		**Number (%)**
The HAT test is inconvenient to use at home	Yes	6 (18.7)
Trouble caregiver at home to assist in doing HAT test	Yes	3 (9.4)
Concerned with the accuracy of HAT test findings	Yes	1 (3.1)
Difficult to understand the reading of HAT test	Yes	2 (6.3)
Afraid of pricking my own finger	Yes	2 (6.3)
Prefer in-person visit with my doctor	Yes	1 (3.1)
Still need to come to clinic for medication	Yes	1 (3.1)
Not applicable	Yes	20 (62.5)

**Table 3:** Steps involved in using HAT kit at home and reported challenges

Difficult/challenging step of using HAT kit at home		Number (%)
Differentiating between pouch 1 and pouch 2	Yes	0 (0.0)
Difficulty in reading small print on the reader	Yes	1 (3.1)
Inserting collector into mixer	Yes	0 (0.0)
Storing the test kit in refrigerator at home (e.g., hygiene issue, space issue, not sure of fridge temperature)	Yes	6 (18.7)
Having to thaw to maximum of 28 °C (e.g., having to use air conditioning)	Yes	6 (18.7)
Having to wait after taking the kit out of fridge including the thaw time	Yes	4 (12.5)
Time needed to perform the test	Yes	1 (3.1)
Fear of getting testing error	Yes	3 (9.4)
Too many steps to remember to complete the test	Yes	9 (28.1)
Do not feel confident to do HAT test at home alone/by myself	Yes	1 (3.1)
Inconveniencing caregiver to help with HAT test at home	Yes	1 (3.1)
Fear of needles	Yes	0 (0.0)
Using Bluetooth to transmit the reading	Yes	14 (43.7)
Not applicable	Yes	11 (34.4)

Overall, participants reported being satisfied with different components of the PTEC HAT Programme. About 87.5% either agreed or strongly agreed with being satisfied with the medical advice that they received through phone consultations. Almost half of the patients (53.1%) either agreed or strongly agreed that they found it convenient to submit their HbA1c measurements to their care team by clicking “submit” button. About 75.0% either agreed or strongly agreed that they found that their care team understood their condition well because they had access to the readings which participants take at home. Lastly, about 46.9% either agreed or strongly agreed that they felt motivated to control their blood sugar better in this program. (Table 4).

**Table 4:** Satisfaction of participants with the PTEC HAT Programme

Satisfaction with using HAT kit at home		Number (%)
Satisfied with the medical advice that I receive through phone consultations	Strongly Disagree or Disagree	0 (0.0)
	Unsure but probably disagree	0 (0.0)
	Unsure but probably agree	4 (12.5)
	Strongly Agree or Agree	28 (87.5)
Find it convenient to submit my HbA1c measurements to my care team by clicking “submit” button	Strongly Disagree or Disagree	7 (21.9)
	Unsure but probably disagree	2 (6.3)
	Unsure but probably agree	6 (18.7)
	Strongly Agree or Agree	17 (53.1)
Find the care team understands my condition well because they have access to the readings which I take at home	Strongly Disagree or Disagree	1 (3.1)
	Unsure but probably disagree	1 (3.1)
	Unsure but probably agree	6 (18.8)
	Strongly Agree or Agree	24 (75.0)
Feel motivated to control my blood sugars better in this program	Strongly Disagree or Disagree	7 (21.9)
	Unsure but probably disagree	1 (3.1)
	Unsure but probably agree	9 (28.1)
	Strongly Agree or Agree	15 (46.9)

While the average of overall visits was similar across both six months pre- and post-enrolment, the average of diabetes-related visits was significantly lower for six months post-enrolment as compared to six months prior. The HbA1c values were comparable for six months before and after the intervention. The average BP (both systolic and diastolic) was lower for six months post-enrolment as compared to six months prior, but this did not reach statistical significance because of the small sub-sample size. (Table 5)

**Table 5:** Comparison of pre- and post- intervention visits and vital parameters of participants

Parameter		Pre-Intervention^1^	Post-Intervention	*P*-value^2^	N (% of total sample)^3^
All cause Visits	Mean (SD)	3.6 (1.6)	3.6 (2.0)	1.00	32 (100.0%)
	Min, Max	1.0, 8.0	1.0, 10.0		
Diabetes related Visits	Mean (SD)	0.8 (0.7)	0.3 (0.6)	**0.01**	32 (100.0%)
	Min, Max	0.0, 2.0	0.0, 2.0		
HbA1c (%)	Mean (SD)	7.1 (0.6)	7.4 (0.8)	0.17	5 (16.0%)
	Min, Max	6.2, 7.7	6.3, 8.4		
Systolic BP (mmHg)	Mean (SD)	130.3 (11.4)	127.1 (10.4)	0.37	12 (38.0%)
	Min, Max	118.0, 154.0	115.0, 149.0		
Diastolic BP (mmHg)	Mean (SD)	76.0 (9.2)	75.2 (7.1)	0.66	12 (38.0%)
	Min, Max	57.0, 90.0	64.0, 91.0		

1: includes all visits before the enrolment for PTEC-HAT Pilot excluding the enrolment visit

2: *P*-value computed using paired t-test

3: Missingness is due to unavailability of electronic medical record data (and not due to patients not completing home HbA1c testing) for the post-intervention period. For the purpose of generation of summary statistics, the sample was limited to the data available at both pre- and post-intervention period

### Qualitative findings (based on open-ended feedback)

Based on open-ended feedback, the most liked feature of the programme was the removal of the need to have a clinic visit and related travel time (N = 6). (Additional File 2).

“*Due to my profession, I do not need to take no pay leave when visiting the doctor. I can do the test at my home at any timing on the day itself*.” (Female, 41 to 50 years).

The next liked feature was the educational videos, which were found to be useful by the patients (N = 4).

“*The test is ok. The duration from the practice day to testing day is far and I could not remember the steps, but the video helped to refresh my memory.*” (Male, 51 to 60 years).

Interestingly, a few participants suggested that HAT kit usage under the PTEC HAT Programme is a good system for a subgroup of patients who are disciplined enough to follow through the home testing steps (N = 3).

“*A great system for disciplined patients. Appreciate all efforts put in by the healthcare providers into the programme*.” (Female, 51 to 60 years).

The most disliked features were the involvement of too many steps in home testing using the HAT kit (N = 5) and the use of Bluetooth to transmit the readings (N = 5).

“*Too many steps, have to follow the video to submit readings. Took about 3 times the duration.*” (Female, 41 to 50 years).

“*Bluetooth pairing is complex. Had to do many rounds to get the final results submitted through*.” (Female, more than 60 years).

Based on open-ended feedback, the most commonly suggested improvements were to streamline the data transmission process (involving Bluetooth) (*N* = 8) and decrease the number of steps involved in the testing process. Other proposed improvements were to have reasonable pricing (*N* = 4), easier to understand instructions (*N* = 4), increase the frequency of testing to help participants remember the steps (*N* = 2), select the right group of patients for using the HAT kit (*N* = 2), etc. (Additional File 2)

## Discussion

Adopting an implementation perspective to bridge the knowledge-practice gap, the findings from this PTEC HAT Programme feasibility pilot support that home HbA1c testing by patients coupled with telemonitoring and teleconsultations by the care team was well-received by the patients with diabetes, highlighting the key perceived benefits, challenges, barriers and suggested improvements. Additionally, participants reported high satisfaction with different components of the PTEC HAT Programme. Our findings will inform the development and implementation of the PTEC HAT Programme on a larger scale with subsequent integration into the usual care practice within Singapore.

All participants in our feasibility pilot were able to complete the home HbA1c test with differing levels of assistance provided by the care team. Also, the majority of the participants appreciated the benefits of self-testing for HbA1c at home. Similar to our findings, a US-based feasibility study reported that patients with type 2 diabetes were able to successfully complete at least one home HbA1c test, but the participants required assistance either via phone or Zoom meeting [11]. While the most common reported error in this study was the inadequacy of the blood sample, our findings highlighted the Bluetooth transmission issue as the central pain point or challenge for the participants [11]. This difference could possibly be explained by the extensive training and education efforts undertaken in our feasibility pilot, which also received positive feedback from the participants and potentially resulted in participants being able to perform the HbA1c test at home. This is supported by findings from a study that reported participants performing home HbA1c testing to report positively on the usefulness of the educational videos and differential test completion failure rate in those who were provided with educational videos versus those who were not [9]. Similar to the challenge related to Bluetooth transmission of readings, a recent systematic scoping review identified “difficulty using technology” as the most common patient-level barrier to using digital health technology [26]. Hence, to increase the adoption of the PTEC HAT Programme in the subsequent implementation scaling phase, it is important to address this barrier and make the transmission of readings effortless and easy. One solution for the implementation scaling phase would be for the study team to explore alternative modes of transferring their readings, for example, via optical character reading (OCR) or manual submission [27]. 

The second most commonly reported challenge was having too many steps to remember to complete the test. The requirement to complete multiple steps accurately in the prescribed manner to be able to get a correct reading was perceived as challenging by the participants as it may have resulted in increased cognitive load for the participants. Cognitive load, defined as the “*effort and mental resources required to complete a task*” [28], is increased by factors such as the number of items to be learned and completed, the novelty and structure of a task and a person’s cognitive ability and prior experience with similar skills [29]. It would be useful to reduce the cognitive load for patients in the implementation scaling phase by providing educational resources developed during this feasibility pilot phase, such as user-friendly instructional videos and accompanying guides to improve compliance and adoption of this intervention.

The most commonly reported benefit of home HbA1c testing in our feasibility pilot was the convenience of receiving the test results and subsequent management by the care team at home without the need to go to the clinic. In concordance with our findings, teleconsultations in primary care setting have been reported to be time-efficient, cost-saving [30] and perceived as convenient, facilitating easy access to healthcare services [31]. Interestingly, a few participants shared that the PTEC HAT Programme would be useful for a selected group of patients with high self-efficacy, self-discipline and digital literacy to follow through the steps of home HbA1c testing, which is aligned with existing literature on the role of self-efficacy and digital literacy in the adoption of digital technology interventions [32]. Overall, patients reported being satisfied with different components of the PTEC HAT Programme. About 87.5% either agreed or strongly agreed with being satisfied with the medical advice that they received through phone consultations. Almost half of the patients (53.1%) either agreed or strongly agreed that they found it convenient to submit their HbA1c measurements to their care team by clicking “submit” button. This is aligned with existing literature whereby participants have reported high satisfaction with performing home HbA1c testing [9]. 

Our results of clinical outcomes were largely aligned with a controlled trial which reported improvement in both HbA1c and BP at the end of 6 months of follow-up. Specifically in this controlled trial, the proportion who achieved HbA1c reduction of 0.5% or more in the intervention group was significantly higher as compared to the control group in this controlled trial. Additionally, the reduction in both systolic and diastolic BP was statistically significant in the intervention group in this controlled trial at 6 months [13]. Hence, in concordance with existing literature, our findings of maintenance of glycaemic control and improvement in both systolic and diastolic BP support the use of home HbA1c testing combined with telemonitoring and teleconsultation for diabetes management and the potential achievement of favourable clinical outcomes. However, we do acknowledge that our findings of HbA1c and BP were not statistically significant which is due to the feasibility nature of current study with limited sample size. Therefore, our findings can be further substantiated by transitioning from this feasibility pilot phase to the implementation scaling phase to manage a larger volume of patients with diabetes in the real-world setting. We reported that while the overall visits were similar across 6 months pre- and post-enrolment, the diabetes-related visits were lower during the intervention period as compared to six months prior, which is aligned with previous studies reporting POC HbA1c testing, reducing manpower costs and clinic visits [8, 33]. 

Our study has several strengths. We adopted a co-development approach with our intended end-users (i.e., the healthcare team in the primary care setting) from an implementation perspective which is reported to facilitate the application of research efforts in the real-world setting at scale [34–36]. Another advantage was the implementation feasibility scope, which meant not adding additional elements (e.g., calling participants back for data collection, etc.) to the usual care of patients with diabetes apart from the alternative care model with home HbA1c being tested. This makes our user experience and effectiveness estimates as close as possible to the estimates expected in the usual care setting when this alternative care model is implemented at scale. We captured both quantitative and qualitative data (in the form of open-ended questions) on the user experience, perceived challenges and benefits of the PTEC HAT Programme, which allowed us to get balanced, triangulated findings. Lastly, we are the first, to the best of our knowledge, to conceptualize and test the operational viability of an alternative care model involving a self-test of HbA1c at home by the patient, combined with the telemonitoring and teleconsultations by the care team in the primary care setting of Singapore, with the potential of widespread implementation in future.

The following are the limitations of our study. Considering the operational feasibility as well as the real-world implementation scope, our data collection was limited to maintain care as usual apart from the intervention itself; hence we did not capture additional self-reported measures to assess improvement in self-care practices, lifestyle changes, as past literature has reported the benefits of a similar intervention to extend beyond positive user experience and improved glycaemic control [13]. Additionally, we did not capture the treatment change or medication adherence data within this feasibility pilot, which may have provided insights into the possible mechanism of improvement or maintenance in HbA1c. However, it is aligned with the implementation feasibility scope of the current study with the primary focus on the experience of conducting home HbA1c testing to inform widespread scaling efforts in the future. Moreover, similar interventions in the past have also reported modest to no differences in treatment changes or medication adherence at the end of such interventions [13]. Another limitation of our study is the relatively small sample size. However, this is not uncommon in an implementation feasibility context and a similar sample size (*N* = 30) has been reported previously by another feasibility study testing the operational viability of POC testing for HbA1c in the primary care setting [7]. 

Following are the practical recommendations from our study. The PTEC HAT Programme, including the home HbA1c testing by patients with diabetes, should be offered to suitable candidates having adequate levels of digital literacy who display the drive for self-management and the ability to perform the home HbA1c test independently or with minimal assistance. After implementing this intervention at scale over a longer duration with a larger sample size of patients with diabetes, the impact of the intervention can be assessed on other relevant outcomes like albuminuria, glomerular filtration rate, presence and progression to retinopathy etc. Since more than half of the patients (59%) in the PTEC HAT Programme feasibility pilot felt it was challenging to do a home HbA1c test, it is recommended that adequate support be provided to the patients in the future to help them overcome these challenges. The conduct of the home HbA1c test itself was perceived to be complicated (e.g., having too many steps, etc.). Hence, the provision of adequate training, supporting educational materials and re-enforcement of training, where needed, via sending of reminder messages with embedded links to training videos, may result in greater compliance and successful completion of home HbA1c testing. Since existing educational videos were found to be useful by the participants, these can be offered as part of the PTEC HAT Programme when implemented at scale. It would be helpful to make manual submission of the home HbA1c reading more acceptable or a default option since the most commonly reported challenge was using Bluetooth to transmit the home HbA1c reading. Lastly, considering the price sensitivity of participants, with only 22% willing to pay for this intervention, it is important to ensure the offered price is acceptable to the patients when integrating the intervention into usual care.

## Conclusion

Adopting an implementation perspective to bridge the knowledge-practice gap, the findings from this PTEC HAT Programme feasibility pilot support home HbA1c testing by patients coupled with telemonitoring and teleconsultations by the care team, and highlight the key perceived benefits, challenges, barriers and possible improvements. Additionally, participants reported high satisfaction with different components of the PTEC HAT Programme. Our findings will inform the development and implementation of the PTEC HAT Programme on a larger scale with subsequent integration into the usual care practice within Singapore.

### Electronic supplementary material

Below is the link to the electronic supplementary material.

Supplementary Material 1

Supplementary Material 2

## Data Availability

The datasets generated and/or analysed during the current study are not publicly available due to confidential nature of the datasets but are available from the corresponding author on reasonable request.

## Abbreviations

BP: Blood Pressure
CLIA: Clinical Laboratory Improvement Amendments
DCP: Diabetes Care Profile
DES-SF: Diabetes Empowerment Scale
DKT: Diabetes Knowledge Test
IFCC: International Federation of Clinical Chemistry and Laboratory Medicine
IT: Information Technology
IVD: In Vitro Diagnostic
HbA1c: Glycated Haemoglobin
HSA: Health Science Authority
MOHT: MOH Office for Healthcare Transformation
NGSP: National Glycohemoglobin Standardization Programme
NHGP: National Healthcare Group Polyclinics
OCR: Optical Character Scanning
POC: Point Of Care
PTEC-HAT: Primary Technology Enhanced Care Home HbA1c Testing
SD: Standard Deviation
