# Supplementary material for: Primary Technology Enhanced Care Home HbA1c Testing (PTEC HAT) programme: a feasibility pilot study in Singapore
Source: BMC Prim Care. 2024 Apr 23;25:127. doi: 10.1186/s12875-024-02373-w (PMC11040893; doi:10.1186/s12875-024-02373-w)
Supplement: Supplementary file 1 — Supplementary Material 1 [file 12875_2024_2373_MOESM1_ESM.pdf]

USER EXPERIENCE SURVEY (PTEC-HAT PILOT)

Q1.	Do you feel there are any benefits of using A1C test kit at home?
	- Yes<br>- No

Q2.	Based on your experience using A1C test kit at home, please select the benefits applicable to you (you can select more than 1):	
	a.	No need to visit the clinic
	b.	Save transport related cost and time
	c.	It is more convenient
	d.	Do not have to trouble my caregiver to assist me with clinic visit
	e.	I am more aware of my readings
	f.	I am more motivated to manage my diabetes
	g.	Tele-consults with care team to get feedback on my A1C test readings
	h.	Not Applicable

Q3.	Do you feel there are any barriers to using A1C test kit at home?
	- Yes<br>- No

Q4.	Based on your experience using A1C test kit at home, please select the barriers applicable to you (you can select more than 1):	
	a.	The A1C test is inconvenient to use at home
	b.	Trouble caregiver at home to assist in doing A1C test
	c.	Concerned with the accuracy of A1C test findings
	d.	Difficult to understand the reading of A1C test
	e.	Afraid of pricking my own finger
	f.	Prefer in-person visit with my doctor
	g.	Still need to come to clinic for medication
	h.	Think condition will worsen if wait too long between in-person visits
	i.	Not Applicable

Q5.	Do you feel it is difficult/challenging to do A1C test at home?
	- Yes<br>- No

Q6.	Which of the following steps of A1C test do you find most difficult/challenging (you can choose more than 1 option)?	
	a.	Differentiating between pouch 1 and pouch 2
	b.	Difficulty in reading small print on the reader
	c.	Inserting collector into mixer
	d.	Storing the test kit in refrigerator at home (e.g., hygiene issue, space issue, not sure of fridge temperature)
	e.	Having to thaw to maximum of 28 degrees C (e.g., having to use air conditioning)

	f.	Having to wait after taking the kit out of fridge including the thaw time
	g.	Time needed to perform the test
	h.	Fear of getting testing error
	i.	Too many steps to remember to complete the test
	j.	Do not feel confident to do A1C test at home alone/by yourself
	k.	Inconveniencing your caregiver to help with A1C test at home
	l.	Fear of needles
	m.	Using Bluetooth to transmit the reading
	n.	Not Applicable

Q7.	What do you like or dislike about using A1C test kit at home? Please also share any other feedback that you may have. -----------------------

Q8.	Based on your experience using A1C test kit at home to monitor your diabetes, will you be willing to pay for this service of home HbA1c monitoring along with tele-consult with your care team in future?
	- Yes<br>- No<br>- Don’t know

Q9.	Considering your experience doing A1C test at home, how likely would you be to recommend A1C test at home to a friend or family member or colleague?									
0	1	2	3	4	5	6	7	8	9	10

Very Unlikely — Very Likely

Q10.	If A1C test kit for home monitoring is offered to patients attending polyclinics in future, what can be improved to increase the uptake and adoption? -----------------------

**Patient satisfaction**

Now, after each statement, circle the number that best describes how often you feel that way. Please circle only one number for each item.

**Items**	**Choose the most appropriate response**	
Q11. I am satisfied with the medical advice that I receive through phone consultations	Strongly disagree	1
	Disagree	2
	Unsure but probably disagree	3
	Unsure but probably agree	4
	Agree	5
	Strongly agree	6
Q12. I find it convenient to submit my hbA1c measurements to my care team by clicking the "Submit" button on the machine.	Strongly disagree	1
	Disagree	2
	Unsure but probably disagree	3
	Unsure but probably agree	4
	Agree	5
	Strongly agree	6
Q13. I find that the care team understands my condition well because they have access to the readings which I take at home.	Strongly disagree	1
	Disagree	2
	Unsure but probably disagree	3
	Unsure but probably agree	4
	Agree	5
	Strongly agree	6
Q14. I feel motivated to control my blood sugars better in this program.	Strongly disagree	1
	Disagree	2
	Unsure but probably disagree	3
	Unsure but probably agree	4
	Agree	5
	Strongly agree	6
