# Supplementary material for: Primary Technology Enhanced Care Home HbA1c Testing (PTEC HAT) programme: a feasibility pilot study in Singapore
Source: BMC Prim Care. 2024 Apr 23;25:127. doi: 10.1186/s12875-024-02373-w (PMC11040893; doi:10.1186/s12875-024-02373-w)
Supplement: Supplementary file 2 — Supplementary Material 2 [file 12875_2024_2373_MOESM2_ESM.pdf]

SUPPLEMENTARY TABLES

Supplementary Table 1. Open-ended feedback on PTEC HAT Pilot Programme experience (N=33)

Feedback (open-ended)	Frequency
**Likes**	
Like the HAT kit	1
No need to visit the clinic	6
Instructional videos are useful	4
Good system for disciplined patients	3
**Dislikes**	
Too many steps to conduct the test	5
Bluetooth transmission issues	5
HAT kit storing conditions at home	2
Need to remember the steps of test	4
Lack of human touch	1
Inconvenient	2
Time consuming	1
Not suitable for non-tech savvy	3
User guide not very user friendly	1
No feedback	7

Supplementary Table 2. Suggested improvements to increase uptake of PTEC HAT Programme in future (N=33)

Suggested Improvement	Frequency
Improve data transmission	8
Decrease the number of steps	5
Price must be reasonable	4
Easier to understand instructions	4
Increase frequency of testing	2
Select right group of patients (e.g., tech-savvy)	2
Include caregiver in training process	1
Still have F2F consults	1
Advertise and reach out to more diabetics	1
Improve packaging of the kit	1
